# Plasma selenium and risk of dysglycemia in an elderly French population: results from the prospective Epidemiology of Vascular Ageing Study

**DOI:** 10.1186/1743-7075-7-21

**Published:** 2010-03-18

**Authors:** Tasnime N Akbaraly, Josiane Arnaud, Margaret P Rayman, Isabelle Hininger-Favier, Anne-Marie Roussel, Claudine Berr, Annick Fontbonne

**Affiliations:** 1 Inserm, U888, F-34093, Montpellier, France; Université Montpellier 1, Montpellier, France; 2 Department of Epidemiology and Public Health University College London, London, UK; 3 Grenoble University Hospital, Department of Biochemistry, Toxicology and Pharmacology, Grenoble, France; 4 Inserm, U884, Grenoble, F-38000, France; University Grenoble, Grenoble, France; 5 Nutritional Sciences Division, Faculty of Health and Medical Sciences, University of Surrey, Guildford, UK; 6 IRD, UMR204, Montpellier, France

## Abstract

**Background:**

A preventive role of selenium on the risk of diabetes has been reported and ascribed to the "insulin-like" activity of selenium and the antioxidant properties of the selenoenzymes. By contrast, data from cross-sectional studies and clinical trials have suggested an adverse effect of high selenium status and selenium supplementation on type-2 diabetes risk. Given these controversial results, we investigated prospectively the relationship between baseline plasma selenium concentration and occurrence of dysglycemia (impaired fasting glucose or type 2 diabetes) in an elderly French cohort.

**Methods:**

The Epidemiology of Vascular Ageing (EVA) study (n = 1389, 59-71 years) is a 9-year longitudinal study in which, fasting plasma glucose was measured at baseline, 2, 4 and 9 years. Analyses were performed on 1162 participants with complete data.

**Results:**

At baseline plasma selenium mean levels were 1.08 (0.21) μmol/l in men and 1.10 (0.20) μmol/l in women. During the 9-year follow-up, 127 cases of dysglycemia occurred. A significant interaction was found between plasma selenium and sex. Risk of dysglycemia was significantly lower in men with plasma selenium in the highest tertile (T3:1.19-1.97) compared to those in the lowest tertile (T1:0.18-1.00) [HR = 0.48 (0.25-0.92)], but no significant relationship was observed in women. After controlling for socio-demographic factors, lifestyle factors, cardiovascular diseases, body mass index, hypertension and lipid profile, plasma selenium remained marginally significantly associated with occurrence of dysglycemia in men [T3 *vs*. T1, HR = 0.50 (0.24-1.04)] and unrelated in women.

**Conclusions:**

This prospective study suggests a sex-specific protective effect of higher selenium status at baseline on later occurrence of dysglycemia.

## Background

Type 2 diabetes is a common burden in the elderly [1]. In this context, identifying nutrients that could help reduce diabetes in an elderly population is a worthy public-health aim.

Selenium is an essential trace element. Its importance is underlined by the fact that it is the only trace element to be specified in the genetic code - as selenocysteine. Selenium is a key component of several functional selenoproteins [e.g., glutathione peroxidases (GPx), thioredoxin reductases, iodothyronine deiodinases and selenoprotein P] that protect tissues and membranes from oxidative stress and control the cell redox status [2]. Evidence from *in vivo *and *in vitro *studies suggests that selenium could enhance insulin sensitivity by mediating insulin-like actions [3,4].

Results from human studies on selenium and diabetes are conflicting. Two studies found lower serum selenium concentrations in diabetic patients than in control subjects [5,6] while in the Health Professionals Follow-up Study, toenail concentrations were lower in diabetic men than in non-diabetic controls [7]. By contrast, higher serum selenium concentration was associated with a higher prevalence of diabetes [8,9], higher fasting plasma glucose and glycosylated hemoglobin levels [9] in the National Health and Nutrition Examination Survey [8,9]. Randomized trials again showed discordant findings: in the SUVIMAX trial, despite positive correlations between plasma selenium and plasma glucose both at baseline and at the end of the follow-up, no effect of supplementation with a mixture of antioxidants, including 100 μg/day selenium, on plasma glucose levels was found after 7.5 years of follow-up, was found [10]. A recently-published analysis of Nutritional Prevention of Cancer Trial data showed that supplementation with 200 μg/d selenium as high-selenium yeast for 7.7 years increased the risk of self-reported type 2 diabetes [11].

Given these controversial results, we investigated prospectively the relationship between baseline plasma selenium concentration and occurrence of diabetes or impaired fasting glucose in a cohort of healthy elderly French men and women followed up for 9 years.

## Methods

### Subjects

The EVA (Epidemiology of Vascular Ageing) study is a 9-year follow-up longitudinal study primarily aimed at studying cognitive impairment and vascular ageing [12,13]. At baseline (EVA0, 1991-1993), 1389 volunteers (574 men and 815 women, mean age 65 yr) residing in the town of Nantes (Western France) were recruited. The subsequent follow-up waves with biochemical measurements were EVA2 (2-year follow-up, n = 1272), EVA3 (4-year follow-up, n = 1188) and EVA6 (9-year follow-up, n = 781). The study protocol was approved by the Ethical Committee of the University Center Hospital of Kremlin-Bicêtre, France. Signed, informed consent was obtained from all participants at enrolment.

The present analyses relate to the 1162 participants who were normoglycemic [fasting blood glucose (FBG) ≤ 6.1 mmol/L] and not using anti-diabetic drugs at baseline, and for whom plasma selenium measurements were available. Characteristics of the 635 participants who completed the 9-year follow-up were compared to those of the 527 who did not, of whom 101 had died. The latter were more likely to be current or former smokers (43.4% *vs*. 37.5%, *P *= 0.04), to have higher baseline diastolic blood pressure (79.5 ± 1.0 *vs*. 78.2 ± 0.7 mm Hg, *P *= 0.03); there were no other significant differences between the groups.

According to the WHO definition [14], participants with FBG ≥ 7 mmol/L or who used anti-diabetic drugs were defined as diabetic; participants with FBG between 6.1 and 7 mmol/L were considered as having impaired fasting glucose (IFG). Dysglycemia was defined as the presence of IFG or diabetes.

### Methods

A baseline questionnaire recorded information on sex, age, education (≤ primary school/> primary school), smoking habits (current/ex-smoker/non-smoker), alcohol intake (≥ 20 ml/< 20 ml per day), use of lipid-lowering drugs, use of antihypertensive drugs and history of cardiovascular diseases (CVD) (myocardial infarction, angina pectoris and stroke). Height and weight were measured and body mass index (BMI) was calculated as weight (kg) divided by height squared (m^2^). Two independent measures of systolic and diastolic blood pressure were taken with a digital electronic tensiometer after a 10-minute rest, from which the means were calculated.

Blood samples were drawn between 8.30 am and 9.30 am after a 12-hour fast. Analytical procedures for the determination of glucose, LDL-cholesterol, HDL-cholesterol and triglycerides, thiobarbituric acid reactive substance concentrations (TBARs) and erythrocyte GPx activity (only measured at EVA2) have been described elsewhere [13]. Plasma selenium was determined using electrothermal atomic absorption spectrometry (Perkin Elmer 5100 ZT, Norwalk, CT, USA) [15].

### Statistical analysis

Plasma selenium was categorized by tertiles. Survival analyses by an actuarial method were used to assess the occurrence of dysglycemia according to tertile of plasma selenium at baseline [S(t)]. The association between plasma selenium tertile and onset of dysglycemia was determined by Cox proportional hazards regression models. The proportional hazards assumption was verified by adding a time-dependent variable to the model. Additionally to age, analyses were adjusted for education, smoking habits, alcohol intake, LDL-cholesterol, HDL-cholesterol, triglycerides, use of lipid-lowering drugs, systolic and diastolic blood pressure, use of antihypertensive drugs, history of CVD and BMI. All interactions between plasma selenium and other variables were tested. The presence of a significant interaction with sex (p < 0.001) led us to carry out analyses separately in men and women.

To explore whether relationships with plasma selenium might be related to a redox effect of selenoproteins, analyses were performed to assess the relationship between markers of antioxidant status i.e. TBARs and erythrocyte GPx activity and risk of dysglycemia. The level of statistical significance was set at p = 0.05, and marginally significance was defined by 0.05> p < 0.10. Statistical analyses were performed using SAS software version 9.1 (SAS Institute Inc., Cary, North Carolina, USA).

## Results

The median and range values for each tertile of plasma selenium were 0.90 μmol/L (0.18-1.00) for T1 (<33^rd^), 1.09 μmol/L (1.01-1.18) for T2 (≥ 33^rd ^<66^th^), 1.32 μmol/L (1.19-1.97) for T3 (≥ 66^th^). Plasma selenium concentrations did not differ significantly between men and women. Factors associated with plasma selenium are shown separately for men and women in Additional file 1. No significant association was found between selenium tertile and age, education, alcohol intake, LDL cholesterol, triglyceride, use of hypertensive drugs, blood pressure and BMI in either gender. While in women, smoking habit was not associated with selenium status, unexpectedly, men who were current or former smokers were more likely to be in the top tertile of selenium. Regarding blood lipids, HDL-cholesterol increased significantly across selenium tertiles in women but not in men. While LDL-cholesterol and triglyceride were not associated with Se, users of lipid-lowering drugs were more likely to have high levels of selenium both in men and women.

During the 9-year follow-up, 127 new cases of dysglycemia occurred of which 15.8% were in men and 7.8% in women (p < 0.0001), including 29 cases of type 2 diabetes. Among them, 64 cases (including 9 of diabetes) occurred during the first two years of follow-up, 40 (including 10 of diabetes) occurred between EVA2 and EVA3, and 23 (including 10 of diabetes) occurred between EVA3 and EVA6.

**Figure 1:**
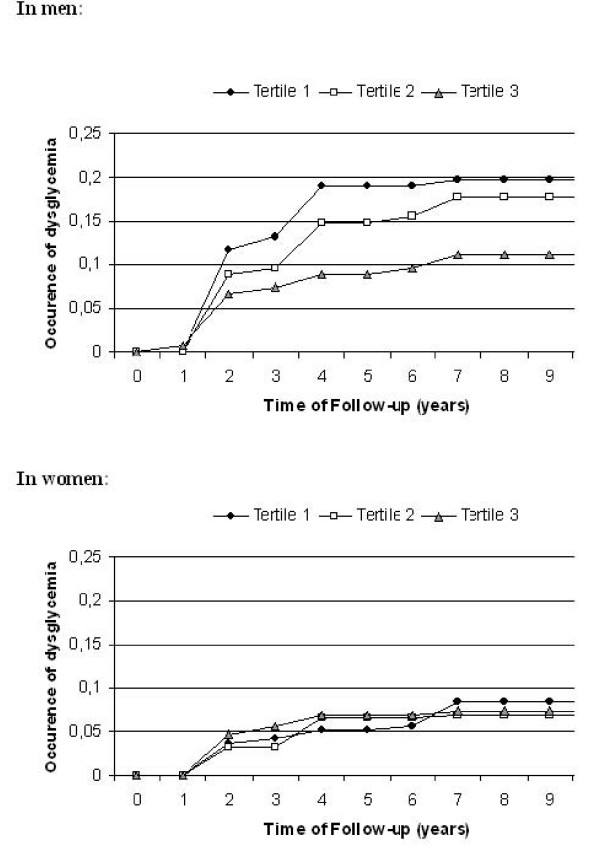
**Occurrence of dysglycemia by tertile of plasma selenium in men (a) and in women (b)**. Tertile 1: 0.18-1.00 μmol/L (median value = 0.90 μmol/L). Tertile 2:1.01-1.18 μmol/L (median value = 1.09 μmol/L). Tertile 3: 1.19-1.94 μmol/L (median value = 1.29 μmol/L). Black circle: Tertile 1; White square: Tertile 2; Grey tangle: Tertile 3.

Occurrence of dysglycemia according to baseline tertile of plasma selenium showed that in men, the lower the tertile (T), the greater the occurrence of dysglycemia (Figure 1a) (results from the log rank test: T1 vs. T3: p = 0.05, T1 vs. T2: p = 0.65, T2 vs. T3: p = 0.13), whereas in women, the occurrence of dysglycemia was comparable in all three tertiles (Figure 1b). Analyses of the relationship of plasma selenium tertile and other population characteristics to the onset of dysglycemia were carried out separately for men and women. Results of unadjusted Cox models (Additional file 2) confirmed that risk of dysglycemia was significantly lower in men with plasma selenium in T3 compared to those with plasma selenium in T1 [T3 *vs*. T1, hazard ratio (HR) = 0.48 (95% confidence interval: 0.25-0.92)] but this was not the case in women [T3 *vs*. T1, HR = 0.89 (0.46-1.72)]. These associations were not much attenuated after adjustment for socio-demographic factors (age, education), smoking, alcohol consumption, health parameters (BMI, diastolic and systolic blood pressure, use of hypertensive drugs, LDL-cholesterol, HDL-cholesterol, triglycerides, CVD history, use of lipid-lowering drugs), with a marginally significant association remaining in men [T3 *vs*. T1, HR = 0.50 (0.24-1.04)] but not in women [T3 *vs*. T1, HR = 1.13 (0.55-2.32)].

In supplementary analyses, we assessed the relationship between markers of antioxidant status - TBARs and GPx activity - and the risk of dysglycemia in participants for whom these markers were available (n = 846). After adjustment for potential confounders, we showed that none of the oxidative stress markers was significantly associated with the risk of developing dysglycemia in either sex over the 9-year period [for erythrocyte GPx activity (by 1UI/I), HR = 0.99 (0.96; 1.02), p = 0.57 in men and HR = 1.02 (0.99; 1.05), p = 0.19 in women and for TBARs (by 1 μmol/L), HR = 1.79 (0.78-4.07) p = 0.17 in men and 1.18 (0.53-2.62), p = 0.69 in women].

## Discussion

Our results showed that for French elderly males, having plasma selenium concentrations in the top tertile of the population distribution (1.19-1.97 μmol/L) was significantly associated with a lower risk of developing dysglycemia over the following nine years. This association remained marginally statistically significant after taking into account socio-demographic and lifestyle factors, CVD history, BMI, blood pressure, HDL-cholesterol, LDL-cholesterol and triglycerides. Plasma selenium was not associated with risk of dysglycemia in women, where only high systolic blood pressure, high BMI, alcohol intake, use of lipid-lowering drugs and low HDL-cholesterol were related to the incidence of dysglycemia.

Epidemiological studies that have investigated specifically the association between selenium and diabetes are scarce. Two case-control studies conducted in Europe on a limited number of subjects showed significantly lower selenium concentrations in adult patients with type 2 diabetes compared to controls [5,6]. Similar findings from a cross-sectional analysis of the Health Professionals Follow-up Study showed lower toenail selenium concentrations among men with diabetes (with or without CVD) than among healthy control participants [7]. However, such studies do not allow us to determine whether low selenium was a cause or a consequence of the disease process. Indeed, in inflammatory conditions, decreased selenoprotein expression - especially of the plasma protein, selenoprotein P - is known to occur and can account for the lower plasma selenium concentration observed in such states [16]. A large cross-sectional analysis within the US Third National Health and Nutrition Examination Survey (8,876 adults, ≥ 20 years of age) showed that the highest quintile of serum selenium (≥ 137.66 ng/ml = 1.74 μmol/L) had a significantly increased prevalence of diabetes compared to those in the lowest quintile (<111.62 ng/ml = 1.41 μmol/L), though rather surprisingly, there was no clear dose-response pattern in the three middle quintiles [8]. The recent report from Laclaustra et al. confirms those findings i.e. an increased risk of type 2 diabetes associated with serum selenium concentration in the US middle-aged population (917 adults, ≥ 40 years of age) [9].

Randomized controlled trials have also shown conflicting results. In the SUVIMAX trial, no effect of combined supplementation with antioxidants, including selenium (100 μg/day as high-selenium yeast) was observed on fasting plasma glucose after 7.5 years of follow-up, despite a positive association between glycemia and selenium concentration at baseline in the whole population [10]. However, the SUVIMAX trial was a multi-antioxidant supplementation trial and it is impossible to isolate the effect of selenium from that of other antioxidants administered. More recently, the Selenium and Vitamin E Cancer Prevention Trial (SELECT) that recruited 35,533 men from the United States, Canada, and Puerto Rico with a median follow-up of 5.5 years, reported a small (though non-significant) increased risk of type 2 diabetes in those supplemented with selenium (200 μg/d from *L*-selenomethionine) rather than placebo [RR = 1.07 (0.94-1.22), p = 0.16] [17]. The effect of long-term selenium supplementation as a single nutrient on the incidence of type 2 diabetes was also investigated by Stranges et al. [11]. Their secondary analysis of the Nutritional Prevention of Cancer randomized trial showed a higher cumulative incidence of type 2 diabetes during 7.7 years of follow-up in participants receiving selenium (200 μg/day as high-selenium yeast) than in those receiving placebo. Interestingly, when results were examined by tertile of baseline plasma selenium, the increased risk was only observed in the top tertile (mean baseline plasma selenium >1.54 μmol/L). It should be noted that the median concentration of selenium in the highest tertile of our study was 1.32 μmol/L, equivalent to their lowest tertile (≤ 1.33 μmol/L). It is of course well known that selenium intakes in France, and more generally in Europe, are considerably lower than in the United States [18,19]. Thus, it is possible that the difference between our results and those of Stranges could be partly explained by a selenium-status effect. Another point for consideration is that in this secondary analysis of the Nutritional Prevention of Cancer trial, the diabetes cases were not identified through measurement of fasting blood glucose, but were based on self-report and medical records. To our knowledge, our study is the first prospective observational study to show that healthy elderly men with baseline plasma selenium between 1.19-1.97 μmol/L had a significantly lower risk of developing dysglycemia over a 9-year follow-up period compared to those with plasma selenium concentration below 1.00 μmol/L. This suggests that optimal levels of selenium may reduce the risk of developing hyperglycemia or type 2 diabetes in men.

The mechanism by which selenium may protect against dysglycemia in the present study is unclear but may involve the selenoenzymes, the activity of which would certainly not be optimal at the plasma selenium concentration range of the bottom tertile in our study (0.18-1.00 μmol/L) [2]. The fact that markers of antioxidant status available in our study - TBARs and erythrocyte GPx activity - were not associated with the risk of dysglycemia may suggest that functions of selenoproteins other than those of antioxidants are more important in this context.

Selenium is known to be a trace mineral with a narrow range of optimal intake and considerable inter-individual variability in terms of metabolic sensitivity [20,21] which is at least partly due to the existence of polymorphisms in selenoproteins [22]. Several plausible mechanisms underlie both the potential beneficial and adverse effects of selenium on diabetes [23]. One of the hypotheses attributes the anti-diabetic mechanism of selenium species to their insulin-like properties. This beneficial effect has been observed with selenate and to a lesser extent with selenite and selenomethionine [3]. Experiments on rat tissues suggest that selenate treatment induces increased phosphorylation of the insulin-receptor beta subunit and of other downstream proteins of the insulin-signaling pathway [3]. The anti-diabetic properties of selenium species would lead to inhibition of the insulin signal, antagonizing protein tyrosine phosphatase 1 B (PTP1B). This protein, in normal physiological conditions, is involved in the termination of insulin signalling and exerts a lipogenic effect. However these potential mechanisms suggested by animal studies were observed with high doses of selenate which are toxic in humans. Other experimental studies have investigated the potential pro-diabetic mechanisms of selenium [24-27]. Development of insulin resistance has been described in mice over-expressing GPx1 [24]. Furthermore, it has been suggested that long-term selenium supplementation at high doses may increase the risk of obesity and diabetes by maintaining excessive GPx1 activity that may alter the physiological inhibition mechanism of PTP1B resulting in permanent inhibition of insulin signalling and activation of the lipogenic pathway [24-26].

The reason we observed a protective effect of selenium in men but not in women is not clear. Differences in risk factors in men and women have been reported for a number of diseases including type 2 diabetes [28,29]. Our results clearly show that men are more at risk of diabetes than women as shown by the higher percentage of men having known risk factors at baseline, i.e. smoking, drinking > 20 ml alcohol/day, higher BMI, blood pressure, FBG, plasma triglycerides and lower HDL-cholesterol. This sex effect might be attributed to the better overall antioxidant status of women: we have previously reported in the EVA study that women had higher erythrocyte vitamin E and total carotenoid concentrations than men [30]. Finally, a considerable number of studies have provided evidence that males and females handle selenium differently [31]. Studies have shown that men and women respond differently to dietary selenium with respect to selenoprotein P and GPx4 [32,33]. Evidence of sex-specific differences also comes from animal experiments: sexually dimorphic expression patterns of a number of selenoproteins - GPx1, type I deiodinase and selenoprotein P - have been found in liver and kidney of young and old mice [34]. In the light of such sex-specific findings, it is perhaps not surprising that we saw an association between selenium tertile and subsequent risk of dysglycemia in men but not in women. However, given the fact that the US Third National Health and Nutrition Examination Survey also showed a sex specific association with diabetes, but in the other direction, i.e. with a deleterious effect of high selenium in men but not in women [8], other studies are clearly needed to understand the underlying mechanism of the sex specificity in the selenium-dysglycemia relationship.

Our study has limitations. The EVA study included volunteers with higher educational status and higher incomes than most elderly French. However this should not affect the relationship between plasma selenium and dysglycemia. More importantly, for reasons of statistical power (only 29 incident cases of diabetes), we included IFG in the dysglycemia definition. IFG indicates a risk for diabetes, but we cannot exclude the possibility that fasting glucose may have reverted to normal in some participants which would have resulted in an over-diagnosis of dysglycemia. However, this would have weakened rather than strengthened the true association between selenium and dysglycemia. Another limitation is that data on intake of selenium supplements were not available in our study; this means we cannot know whether subjects with low plasma selenium level at baseline might have taken selenium supplements after the baseline. However in the 1990s in France, as compared to the US or the UK, very few people took selenium supplements making it less probable that the relationship observed between blood selenium and onset of dysglycemia would be biased by not taking selenium supplementation into account. Finally, another limitation is the high rate of attrition in this cohort. Participants lost to follow-up had lower baseline selenium concentrations. This has probably resulted in an underestimation of the incidence of dysglycemia and consequently decreased the power of the study. Despite these limitations, we observed a significantly lower risk of dysglycemia in men in the highest selenium tertile compared to those in lower tertiles which remained borderline significant after adjusting for a large range of potential confounders."

## Conclusions

In conclusion, our findings added to those of others clearly underline the need for further studies. We need to identify the optimal range of selenium status and intake that will minimize potential adverse effects on glucose metabolism while optimizing type 2 diabetes prevention. This may allow us to target a population that might benefit from selenium supplementation.

## Competing interests

The authors declare that they have no competing interests.

## Authors' contributions

CB designed the study; JA, AMR contributed to the data collection; TNA conducted the statistical analyses and co-wrote the initial and final drafts; TNA, JA, MPR, IHF, AMR, CB and AF co-wrote the final draft.

## Authors' information

TNA: PhD, Research fellow at Inserm U888, Montpellier, France and Honorary Research position at the Department of Epidemiology and Public Health, University College London, London, UK;

JA: PhD, Senior Researcher at Inserm, U884 and the Department of Biochemistry, Toxicology and Pharmacology, Grenoble, France;

MPR: D.Phil, R.P.H.Nutr, Professor at Nutritional Sciences Division, Faculty of Health and Medical Sciences, University of Surrey, Guildford, UK;

IHF&AMR: PhD, Senior Researcher at Inserm, U884, and Grenoble University, Faculty of Biology, Medicine and Pharmacy, Grenoble, France;

CB: MD PhD, Senior Researcher at Inserm U888, University Montpellier 1 and Gui de Chauliac hospital, Montpellier, France;

AF: MD PhD, Senior Researcher at IRD UMR204, Montpellier, France.
